# Atopic Dermatitis: Background, Objectives and Future Perspectives (Superresponders)

**DOI:** 10.3390/life12081192

**Published:** 2022-08-04

**Authors:** Ricardo Ruiz-Villaverde, Javier Domínguez-Cruz, Francisco J. Navarro-Triviño, Manuel Galán-Gutiérrez, Jose Carlos Armario-Hita, Jose Juan Pereyra-Rodriguez

**Affiliations:** 1 Dermatology Department, Hospital Universitario San Cecilio, H.U. San Cecilio, Avda. Conocimiento 33, 18016 Granada, Spain; 2 Dermatology Department, Hospital Universitario Virgen del Rocío, 41013 Sevilla, Spain; 3 Dermatology Department, Hospital Universitario Reina Sofia, 14004 Córdoba, Spain; 4 Dermatology Department, Hospital Universitario Puerto Real, 11510 Cádiz, Spain

In this Special Issue entitled *Atopic Dermatitis: New Perspectives*, we have tried to collect research of special interest related mainly to the incorporation of pathophysiological aspects and therapeutic novelties in this regard.

Pereyra et al. [1] carry out a systematic review and network meta-analysis (NMA) in which they carry out a short-term evaluation of the short-term efficacy and safety of biological molecules and small molecules that have recently been incorporated into our therapeutic arsenal. Upadacitinib and Abrocitinib are the drugs with the highest efficacy, both in monotherapy and in association with TCS. However, they were also those associated with the highest risk of adverse effects, showing monoclonal antibodies’ better safety profile.

Atopic dermatitis (AD) has scarcely studied comorbidities, and among them is the impact on the function and sexual health of patients who suffer from it. Linares-Gonzalez et al. [2] establish that unlike other psychological comorbidities such as anxiety and depression, sexual dysfunction is a field scarcely explored in the literature. This sexual dysfunction focuses on the male sex in large population studies and in clinical diagnoses without exploring it through specific and validated questionnaires in this regard.

Is there any infectious compromise that can worsen the clinical patterns of AD? Navarro-Triviño et al. [3] present a study of sensitization to Malassezia furfur in patients with head and neck AD. It is important to know the differential diagnosis and to approach the study correctly. Sensitization to Malassezia furfur may be one of the main reasons, especially in patients being treated with dupilumab.

The intrinsic relationship linking AD and allergic contact dermatitis is explored by Nemeth et al. [4], and in their study they establish that the rate of AD in the PCHS group and the rate of Preservative contact hipersensitivity (PCHS) in the AD group is remarkable; thus, the role of PCHS should be highlighted in the topical therapy and in the prevention of possible AD exacerbations.

Finally, Ayen-Rodriguez et al. [5] complete this issue by expanding the systematic review and network meta-analysis initially carried out by Pereyra et al. [1], but with much more limited data in the evaluation of efficacy and safety at 52 weeks. The results of the present SR may provide us with a useful basis for the preparation of management guidelines for the use of a new generation of therapies in moderate to severe atopic dermatitis.

However, we wonder what future lines of research are potentially developable from a clinical point of view in AD.

Several immune-mediated skin diseases, including psoriasis, atopic dermatitis (AD), and hidradenitis suppurativa, are therapeutically managed with biologic and non-biologic immunosuppressive and immunomodulatory drugs. One of the most relevant issues in current clinical research is the identification of patients who present a rapid and higher rate of response to biological treatments in comparison to the general population, also known as super responders (SR).

Little scientific evidence to explain the behavior and clinical characteristics of these patients has been published thus far. Its characterization could be important to identify the profile of patients that will respond efficiently to biological treatments.

Among all, psoriasis is the area in which this concept has been worked on the most, where rapid skin clearance is the gold-standard. Patients with a rapid treatment response and achieving an exceptional improvement on their clinical features are so-called SR. Moreover, this concept is not an exclusive feature of psoriasis, since it has also been used in severe asthma [6], cancer [7] and cardiac resynchronization therapies [8], among others.

Although there is no consensus on the definition of SR in psoriasis, it has been used in different studies [9,10,11]. In 2020, Reich and collaborators presented a descriptive poster to the *American College of Dermatology Conference* where they defined a population of SR patients to guselkumab [11]. These VOYAGE 1 and 2 sub-analyses defined SR as patients who achieved PASI100 response at week 20 and 28. These patients also presented greater PASI75-90-100 responses than the placebo-controlled arm over 16 weeks. In order to extrapolate their study into our clinical practice, our research group studied and defined as SR a patient who achieve PASI = 0 at weeks 12 and 24 [12]. We did not find differences in the baseline demographic between SR and non-SR. However, SR showed a trend to be younger, more bio-naïve, and presented less disease duration and comorbidities. Our SR patients also achieved greater PASI75-90-100 responses over 52 weeks. In line with this research, the role of SR is currently being studied in terms of response maintenance to guselkumab across the GUIDE study [13].

AD is making its way as an immune-mediated skin disease, inheriting many of the therapeutic approaches of psoriasis, still with its own unique clinical characteristics. The SR concept breaks through with force, although it requires a clearer and more determined positioning.

Herein, we have carried out a literature review to obtain a reliable approach of the SR concept in AD. In addition, we analyze which of the parameters have been used to coin its definition, highlighting the similarities and differences with psoriasis. All studies have dupilumab as the standard-of-care, as it is the only biological treatment for moderate to severe AD approved by the FDA and EMA until 2022.

To our knowledge, SR definition for AD, was first established in the Dutch registry of AD published by Ariëns et al. [14] (2020). The goal of this study was to evaluate 52-week effectiveness and safety of dupilumab in a prospective, multicenter cohort of adult patients with treatment-refractory AD. This group established the three key domains that are then systematically repeated to identify a patient as SR: improvement in signs (EASI score), symptoms (peak pruritus NRS) and QoL (DLQI score).

These criteria have been replicated in all five publications we have reviewed [13,14,15,16,17,18] with slightly differences among them (Table 1). Truly, the SR term has been only used as such by Ariëns [13] and Nettis [15], while the rest of the authors propose the term *clinically meaningful response*. Noticeable, the two studies sharing the same SR definition criteria, showed an important different percentage of SR patients product of the different thresholds used by each group (40% vs. 63.2% [15]).

After analyzing the studies and comparing them with previously published data on psoriasis, different issues stand out:

(a) The time for response evaluation is 20 to 24 weeks in most psoriasis studies, while it is frankly lower (16 weeks) in the 5 AD studies. In contrast, Silverberg et al. [17] suggests that even a period of 52 weeks should be optimal to evaluate a patient as a SR. It would be more accurate to establish a double range of measurement, either by absolute EASI or by relative EASI with two cut-off points: short and medium term, where the super-response maintenance is demonstrated.
(b) For psoriasis, there is only one objective evaluation of effectiveness, using PASI to define a patient as SR. Additional in AD, both NRS pruritus and DLQI scores are contemplated, introducing the concept of (PROs). The goal in psoriasis is achieving absolute PASI = 0, explained by a longer experience and management with the different biological therapeutic approaches, while in AD, we are still in an initial phase, where it seems difficult to call for achieving an absolute EASI = 0.

Hence, we aim to establish a more realistic definition for SR AD patients.

Thus, it is important to settle on a clear definition of a SR patient, either through biomarkers, clinical evaluation, anthropometric features, or disease evolution parameters. Ultimately, this should be linked to establish optimization patterns to achieve greater therapeutic efficiency when dealing with high impact economic drugs on the public health system.

## Figures and Tables

**Table 1:** Research performed regarding SR on Atopic Dermatitis (AD).

Author/Year	SR or CMR	Criteria	Week	Absolute Reduction EASI ≤ 7, N°. (%)
Ariens LFM [9] 2020	SR	EASI 75/peak pruritus NRS 4 points/DLQI 4 points	16	96 (70.6)
Nettis E [10] 2022	SR	EASI 75/peak pruritus NRS 4 points/DLQI 4 points	16	399 (73.7)
Paller AS [11] 2020	CMR	EASI 50/peak pruritus NRS 3 points/DLQI 6 points	16	28 (33)
Silverberg J [12] 2021	CMR	EASI 50/peak pruritus NRS 4 points/DLQI 3 points	16–52	nd
Canonica WG [13] 2022	CMR	EASI 50/peak pruritus NRS 3 points/DLQI 4 points	16	nd

Super responder (SR); Clinically meaningful response (CMR); Eccema Area Severity Index (EASI); Numerical Rating Scale (NRS), Dermatology Life Quality Index (DLQI). nd: Non disposal.
